# Egr1 Is Necessary for Forebrain Dopaminergic Signaling during Social Behavior

**DOI:** 10.1523/ENEURO.0035-22.2022

**Published:** 2022-04-05

**Authors:** Alexandra Tallafuss, Sarah J. Stednitz, Mae Voeun, Anastasia Levichev, Johannes Larsch, Judith Eisen, Philip Washbourne

**Affiliations:** 1 Institute of Neuroscience, University of Oregon, Eugene, OR 97403; 2 Queensland Brain Institute, Brisbane, QLD 4072, Australia; 3 Max Planck Institut für Neurobiologie, Martinsried, D-82152, Munich Germany

**Keywords:** dopamine, preoptic area, social behavior, zebrafish

## Abstract

Finding the link between behaviors and their regulatory molecular pathways is a major obstacle in treating neuropsychiatric disorders. The immediate early gene (IEG) *EGR1* is implicated in the etiology of neuropsychiatric disorders, and is linked to gene pathways associated with social behavior. Despite extensive knowledge of *EGR1* gene regulation at the molecular level, it remains unclear how EGR1 deficits might affect the social component of these disorders. Here, we examined the social behavior of zebrafish with a mutation in the homologous gene *egr1*. Mutant fish exhibited reduced social approach and orienting, whereas other sensorimotor behaviors were unaffected. On a molecular level, expression of the dopaminergic biosynthetic enzyme, tyrosine hydroxylase (TH), was strongly decreased in TH-positive neurons of the anterior parvocellular preoptic nucleus. These neurons are connected with basal forebrain (BF) neurons associated with social behavior. Chemogenetic ablation of around 30% of TH-positive neurons in this preoptic region reduced social attraction to a similar extent as the *egr1* mutation. These results demonstrate the requirement of Egr1 and dopamine signaling during social interactions, and identify novel circuitry underlying this behavior.

## Visual Abstract

**Figure:**
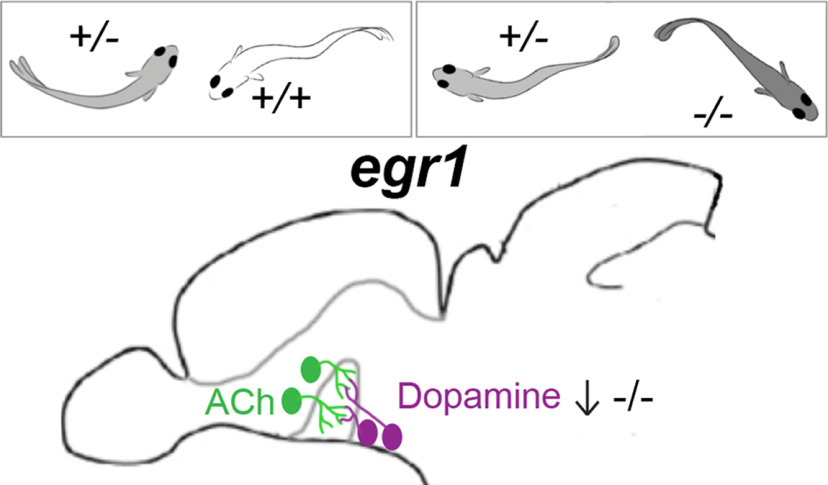

## Significance Statement

*Egr1* is an immediate early gene (IEG) linked to neuropsychiatric disorders, particularly those with social components. However, the role it plays in control of social behavior remains elusive. We found that zebrafish with a mutation in the *egr1* gene present deficits in social approach and orienting behavior. Furthermore, *egr1* mutants have reduced tyrosine hydroxylase (TH) expression, particularly in dopaminergic neurons of the preoptic area (POA) synaptically connected to neurons already identified within a social circuit. Our study provides evidence of a role for *egr1* in establishing social behavior, and adds to our understanding of the underlying circuitry.

## Introduction

Intraspecies interaction, or social behavior, is a widespread phenomenon among animals. It is considered to be beneficial for reasons of predator evasion, reproduction and cooperative foraging (Fernald, 2012). Brain regions, neuronal circuits, as far as they have been characterized, and even genes regulating social behavior are conserved and follow similar basic principles across vertebrates. Therefore, understanding the neuronal circuits and gene networks that underlie social behavior in animal models may provide information pertinent to disorders that compromise human social behavior.

One of the most studied genes with dysregulated expression in disorders with a social component is the immediate early gene (IEG) *EGR1*. In fact, this gene has been evaluated as a biomarker for certain neuropsychiatric disorders (Cattane et al., 2015; Czéh et al., 2016). *EGR1* has been linked to stress-related mood disorders, schizophrenia, and major depressive disorder (Covington et al., 2010; Kimoto et al., 2014). In addition, gene expression studies and gene network modeling have identified *EGR1* as part of a synaptic gene node disrupted in brains of individuals with autism spectrum disorder (ASD; Liu et al., 2016). *EGR1* can perform this nodal function as it is a critical component, similar to other activity-induced transcription factors such as *FOS.* They regulate downstream late-response genes involved in neuronal physiology by dynamically activating a molecular response cascade consisting of a wide range of genes (Duclot and Kabbaj, 2017)

Studies in mouse models also point to a role for *Egr1* in social behaviors. Social defeat stress, a common depression model, reduces *Egr1* expression in the prefrontal cortex (Covington et al., 2010), confirming *Egr1* as biological marker for stress-related depressive disorder. Furthermore, antisense-mediated knock-down of *Egr1* reduces male rat social interaction (Stack et al., 2010), identifying a direct contribution of *Egr1* to social behavior. Conversely, viral-mediated *Egr1* overexpression prevents castration-induced deficits in male rat social interactions (Dossat et al., 2017). Similarly, the optogenetic stimulation of cells in the prefrontal cortex increases *Egr1* expression and induces anti-depressant effects (Covington et al., 2010). In zebrafish, social deprivation results in down-regulation of four genes, including *egr1* (Anneser et al., 2020). In addition, in cichlid fish, *egr1* is specifically induced in the preoptic area (POA) as a response to recognition of social opportunity and ascension to dominance (Burmeister et al., 2005). In response to Egr1 expression, gonadotropin-releasing hormone (GNRH)-expressing neurons undergo physiological changes and exhibit an increase in GNRH mRNA and protein expression (Burmeister et al., 2005). Thus, studies in both the human patient population and vertebrate models suggest a role for *Egr1* in the organization and functioning of brain circuits that support social behavior.

The *Egr1* gene encodes a transcription factor with upregulatory and downregulatory effects on a multitude of target genes. Around 9000 genes, 54% of genes annotated in the Encyclopedia of DNA Elements (ENCODE) project, contain at least one Egr1 binding motif in their promoter region (Duclot and Kabbaj, 2017). Despite this wide-spread potential to bind to promoters, Egr1 shows a bias toward genes involved in regulating synaptic function and plasticity (Duclot and Kabbaj, 2017). For example, Egr1 is recruited to the *PSD-95* promoter in response to NMDA receptor activation (Qin et al., 2015). Also, the presynaptic plasticity gene *Synapsin1* has long been known to be regulated by Egr1 (Thiel et al., 1994). In addition to regulating these synaptic proteins, Egr1 binds to the gene encoding tyrosine hydroxylase (TH), a biosynthetic enzyme for catecholaminergic neurotransmitters (NTs), often involved in response to emotional stress (Papanikolaou and Sabban, 2000; Stefano et al., 2006). Further, the expression of *th* and *egr1* are tightly correlated in response to sensory deprivation in zebrafish (Kress and Wullimann, 2012), suggesting that Egr1 modulates expression of TH-synthesized NTs in an activity-dependent manner. Nevertheless, it remains unclear whether *Egr1* regulates gene expression in specific neural circuits that contribute to social behavior.

Development of robust social behavior was recently described in the zebrafish (Dreosti et al., 2015; Larsch and Baier, 2018; Stednitz and Washbourne, 2020) and a region of the basal forebrain (BF) necessary for this behavior was identified (Stednitz et al., 2018). Here, we examine whether *egr1* plays a role in regulating social behavior in this system using established assays, including a high-throughput virtual biological motion assay (Larsch and Baier, 2018). We find that loss of *egr1* function compromises zebrafish social behavior, but not other behaviors. BF *th* expression is also reduced in *egr1* mutants, with a ∼30% reduction in TH-positive anterior parvocellular preoptic nucleus (PPa) neurons. Chemogenetic ablation of about one third of the identified PPa neurons resulted in social behavior deficits, similar to the reduction observed in *egr1* mutants. Our results suggest that *egr1* is required for expression of *th* in a BF population of dopaminergic neurons necessary for normal social interactions, thereby expanding our understanding of the precise circuitry for social behavior.

## Materials and Methods

### Zebrafish husbandry

All zebrafish embryos, larvae, and adults were raised and maintained at 28.5°C according to standard protocols with a 14/10 h light/dark cycle (Westerfield, 2007). Lines used were AB/Tübingen, *egr1^sa64^*, *Et ^y321^* [*Et(rex2-scp1:gal4ff)y321*], *Et ^y405^* [*Et(scp1:gal4ff)y405*], and *c264Tg(14xuas:nfsB-mCherry)*. The *egr1^sa64^* line, which was generated by ENU mutagenesis during the Sanger Zebrafish Mutation Project (ZMP), was outcrossed to AB/Tübingen for 7 generations before the analysis described. Genotyping was performed for the *egr1^sa64^* allele using a Competitive Allele-Specific PCR SNP genotyping system (KASP, LGC Biosearch Technologies) according to manufacturer’s protocols after behavioral assays and before processing for Western blotting and immunolabeling. Animals with unknown genotypes were excluded from experiments and analysis.

### Behavioral assays

#### Split dyad analysis

Socially-motivated place preference and visually-evoked orienting behavior of 14 d postfertilization (dpf) zebrafish were measured using a split dyad assay (Stednitz and Washbourne, 2020). Roughly size-matched larvae from an incross of *egr1^sa64^* heterozygotes (hets) were placed in paired, isolated tanks (50 mm in length × 20 mm in width × 20 mm in depth) separated by an opaque divider and allowed to habituate for 5 min, then the divider was removed and the animals allowed to interact for an additional 5 min. Both the presocial and social stimulus periods were recorded to determine the baseline exploratory and locomotor behavior. Recordings were obtained from below at 10 fps using a Mightex SME-B050-U camera and illuminated by an overhead white LED panel (Environmental Lights). For numbers of animals and their genotypes see Table 1.

**Table 1:** Animal numbers and genotypes for behavioral experiments

Test	Age (dpf)	Total	+/+	+/−	−/−
Split dyad	14	56	9	36	11
Virtual social	14	75	27	36	12
Virtual social	16	129	38	71	20
Virtual social	18	97	30	49	18
Virtual social (pooled)	14/16	201	172	29	
Predator avoidance	14	126	28	77	21

#### Biological motion social assays

To explore social engagement, we tested social approach using a virtual social assay that is based on detection of biological motion (Larsch and Baier, 2018). The stimuli, which are dots projected below dishes of solitary animals, are followed when they move in bouts with kinetics similar to age matched animals. These assays were performed as described by Larsch and Baier (Larsch and Baier, 2018). Briefly, larvae from an incross of *egr1^sa64^* hets were placed individually in watch glasses filled with facility water. The watch glasses were located in a shallow aquarium on translucent sheets, with a cold mirror and IR LEDs below. A projector, located to the side, projected images onto the translucent sheets by way of the cold mirror. A digital camera with an IR filter was located above the watch glasses. Projector and camera were controlled by bespoke software in Bonsai. Dots of varying sizes were projected intermittently, moving in a synthetic knot shape for a 5-min period. Fish were maintained for 2–3 h in the watch glasses. At each frame, animal and stimulus parameters were streamed to a text file for offline analysis. Fish were tested in facility water at room temperature (25°C). For numbers of animals and their genotypes see Table 1. The numbers for the thigmotaxis and speed analyses are the same as for virtual social at 14 dpf.

#### Chemogenetic ablation experiments

Fourteen days postfertilization larvae from a cross between [*c264Tg(14xuas:nfsB-mCherry)*] and [*Et(scp1:gal4ff)y405*] fish were incubated in the dark for two nights in static facility water with 2.5 μm nifurpirinol (Furanol, JBL), a nitroaromatic antibiotic that is converted into a toxic metabolite by the enzyme nitroreductase. Water flow was restored and larvae were fed during the day. As a control, sibling larvae only expressing GFP, and not the nitroreductase transgene, under the influence of the *Et ^y405^* enhancer trap, were analyzed in parallel. Water flow was restored for a minimum of 1 h before biological motion social assays. A total of 60 animals were tested from two experiments with 28 animals expressing GFP only and 32 animals expressing both GFP and nitroreductase.

#### Predator avoidance tests

Predator avoidance was assessed by measuring visually induced escape responses on presentation of looming stimuli as described previously (Fernandes et al., 2021). Briefly, expanding black dots were projected from below to free-swimming animals using custom scripts in Bonsai to control OpenGL drawing routines. Dots were positioned 10 mm left or right with respect to the center of mass and orientation of each animal at the onset of dot expansion. Dots expanded for 500 ms (15 frames) with a linear increase in diameter to their final size ranging from 0 to 12 mm. Looming stimuli of different sizes were presented left or right of the animals once per minute in random order. A moving grating was presented for 20 s ending 10 s before the presentation of the next loom stimulus to drive larvae toward the center of a dish. At each frame, animal and stimulus parameters were streamed to a text file for offline analysis. Fish were tested at 14 dpf in facility water at room temperature (25°C) for a total of 1 h. For numbers of animals and their genotypes see Table 1.

### *In situ* hybridization (ISH)

RNA ISH was conducted on brain cryostat sections (16 μm) according to standard protocols (Westerfield, 2007) and using the manufacturer recommended protocols for RNAScope (ACD).

### Western blotting

Heads were removed by cutting across the trunk at the level of the heart of anesthetized 15 dpf larvae. Heads were homogenized in RIPA buffer, with the protease inhibitors TLCK, PMSF, and pepstatin. Homogenates were incubated under constant motion at 4°C for 1 h before centrifuging at 12,300 × *g* for 20 min at 4°C. The supernatant was removed and the pellet was resuspended in Laemmli sample buffer with 5% β-mercaptoethanol. Samples were heated at 95°C for 5 min, electrophoresed and blotted to nitrocellulose membranes under standard conditions. Antibodies used are listed in Table 2. Secondary antibodies made in donkey and conjugated to IR dyes 680RD and 800CW (LiCor) were revealed with a LiCor Odyssey.

**Table 2:** Antibody resources for Western blotting

Target	Manufacturer	Catalog #, clone	Dilution	Species	Immunogen	RRID
Syn1/2	Synaptic Systems	106002	1:1000	Rb	Rat peptide, aa 2–28	AB_2619773
TH	Millipore Sigma	AB152	1:5000	Rb	Denatured purified protein from rat	AB_390204
β-Actin	Santa Cruz	Sc-47778	1:1000	Ms	Purified chicken protein	AB_2714189
pan MAGUK	NeuroMab	K28/86	1:1000	Ms	Human PSD-95 fusion protein (77–289)	AB_2877192

Rb, rabbit; Ms, mouse.

### Immunolabeling

Immunolabeling of synaptic markers was performed on brain cryostat sections (16 μm) according to standard protocols (Westerfield, 2007). Antibodies used were to Synapsin 1/2 and Gephyrin (Table 3). Immunolabeling of TH and GFP (Table 3) were performed on paraformaldehyde fixed, dissected heads with jaws and dura removed. Permeabilization, blocking and antibody incubations were performed as described previously (Goode et al., 2020). Primary antibodies were revealed using the following secondary antibodies at a concentration of 1:750: Alexa Fluor goat anti-mouse and rabbit IgG1, IgG_2a_, and (H + L), ThermoFisherScientific, coupled to 488, 546, or 633. Images were taken on a Leica SP8 confocal microscope with LasX software with 10×, 20×, or 63× oil immersion objectives.

**Table 3:** Antibody resources for immunolabeling

Target	Manufacturer	Catalog #, clone	Dilution	Species	Immunogen	RRID
Syn1/2	Synaptic Systems	106002	1:250	Rb	Rat peptide, aa 2-28	AB_2619773
Gephyrin	Abcam	Ab32206	1:1000	Rb	Ms Peptide, aa 700-C-term	AB_2112628
TH	Millipore Sigma	AB152	1:500	Rb	Denatured purified protein from rat	AB_390204
GFP	Invitrogen	3E6	1:500	Ms	Recombinant GFP	AB_2313858

Rb, rabbit; Ms, mouse.

### Quantification and statistics

#### Size comparisons

**Figure 1.**
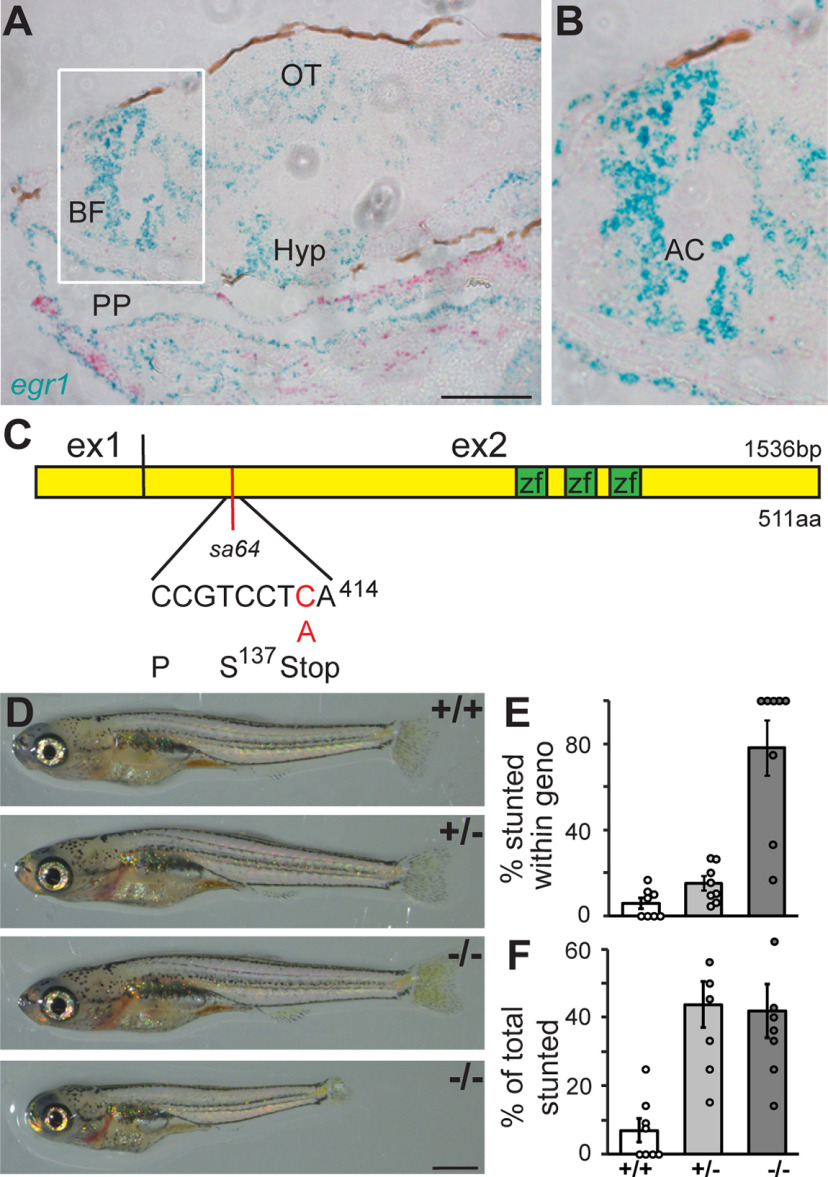
*egr1* is expressed in the forebrain. ***A***, ISH reveals *egr1* expression (blue) in the basal forebrain (BF), optic tectum (OT), hypothalamus (Hyp), and epithelial cells of the pharyngeal pouch (PP) at 6 dpf. *myogenin d* was used as a control probe (red). Anterior to the left. Scale bar: 100 μm. ***B***, Magnification of the BF (white box in ***A***) showing expression surrounding the anterior commissure (AC). ***C***, Exon organization of the *egr1* mRNA, location of the zinc finger domains (zf), and location of the *sa64* mutation (red bar). ***D***, *egr1^sa64^* mutants (−/−) develop a normal body plan with functional swim bladder, but with size deficits (bottom). ***E***, Close to 80% of mutant larvae are stunted (dark gray). ***F***, More than 50% of stunted larvae are also wt (+/+, white) and het animals (+/−, light gray). *N* = 8 clutches, *n* = 248 animals.

Animals were considered “stunted” when they were shorter than a threshold set at 1.5× the SD below average wild-type (wt) size, measured from tip of nose to the end of the trunk (excluding the tail fin). We measured and genotyped 30–32 randomly selected individuals from 8 clutches for the analysis in Figure 1.

#### Split dyad analysis

**Figure 2.**
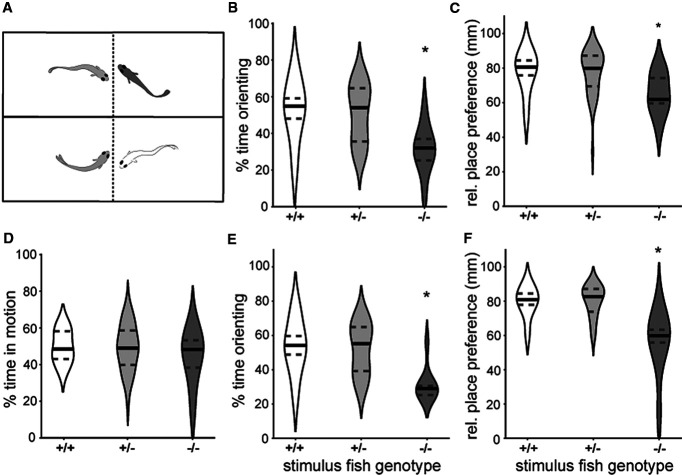
*egr1* mutants are less social than wt larvae. ***A***, Larvae from an incross of *egr1^sa64^* were placed in roughly size-matched split dyads at 14 dpf and tested for social orienting and place preference, with the ability to see a sibling through glass. Genotypes [wt (white), het (gray), mut (dark gray)] were determined after testing. ***B***, *egr1* mutants spent less time orienting at angles between 45° and 90° to the divider. *n* = 56 animals, *p < 0.001 (mmANOVA). ***C***, Relative place preference, as measured from the wall opposite the divider and normalized to the total length, revealed less social engagement for *egr1* mutants. Zero denotes distance furthest from the divider. *p < 0.05 (HSD). ***D***, There was no difference in overall activity measured as percent time in motion. Time orienting at angles between 45° and 90° (***E***) and relative place preference (***F***) for het and wt larvae based on the genotype of the stimulus larvae (stimulus fish genotype) revealed that a deficit in the mutants is detected by the het and wt larvae; *p < 0.001 (mmANOVA). Solid black line represents the median, dotted gray lines represent the upper and lower quartiles.

The percentage of time in motion was calculated as the number of frames in which the animal moved at least one-third of their total body length per frame. Larvae that spent <10% of the experiment in motion were excluded from subsequent analyses. Larvae were genotyped following the experiment as previously described. In separate analyses, data for wt and het animals was pooled and the genotype of the respective stimulus fish was used as the classifier (Fig. 2*E*,*F*). Social interaction is parameterized as the average relative distance from the divider (relative place preference) and the percentage of time spent at 45–90° (% time orienting) using previously described software written in Python (Stednitz et al., 2018). Place preference was calculated as the average relative proximity to the divider across all frames. Behavior was compared statistically using a mixed-model repeated measures ANOVA (mmANOVA) in Python, using genotype as the between-subjects factor and time (presocial and during social stimulus) for each metric as the within-subjects factor.

#### Biological motion social assay and predator avoidance test analysis

Background subtracted, inverted and thresholded avi files were analyzed using scripts in Jupyter as described previously (Larsch and Baier, 2018). The social index (SI) quantifies attraction of the animal toward the biological motion dot stimulus. SI relates the observed animal-dot distance relative to a predicted distance that would result purely from chance encounters with the stimulus. SI was quantified as previously described (Larsch and Baier, 2018) using python scripts: the “real” observed animal-dot distance was calculated for each animal as an average in 5-min chunks (IADr). Next, we shifted the animal trajectories in time relative to the stimulus trajectory by 10 different offsets >60 s and re-calculated a “shifted” animal-dot distance (IADs) for each time shift. Because of the time shift, IADs reflects the dot-animals distance expected by chance encounters. Mean IADs (mIADs) for all time shifts was used to compute SI as (mIADs-IADr)/mIADs. Significance was determined using JMP software. A Tukey–Kramer HSD for pairwise comparisons of least square means was applied to the three genotypes with a *p* threshold of 0.05. Data from within the biological motion social assay was also analyzed for motion and thigmotaxis, as described previously (Larsch and Baier, 2018), and significance was determined using HSD.

#### Western blotting

Images of Western blottings were analyzed using LiCor Image Studio software. We only used intensity data for bands that were within the linear range, i.e., not saturated. Total intensity of bands was divided by the total intensity of in-lane β-actin bands. Resulting intensity ratios were then normalized to the mean of wt larvae. A minimum of two technical replicates were analyzed for four biological replicates. Pools of homogenized heads were of a minimum of ten heads of each genotype. Significance was determined using JMP software. HSD for pairwise comparisons of least square means was applied to the three genotypes with a *p* threshold of 0.05.

#### Synaptic puncta

Cropped sections of 63× images were analyzed in ImageJ using the PunctaAnalyzer plug-in for a region of 77 μm^2^. A minimum of eight images for each genotype was quantified. HSD was applied to the analysis of puncta numbers with a *p* threshold of 0.05.

## Results

### Egr1 influences overall growth but not development

To identify a role for the *egr1* gene in social behavior, we first examined the expression pattern of *egr1* in developing zebrafish larvae by RNAscope ISH at 6 dpf. This developmental period, during which synaptic circuitry remains dynamic (Goode et al., 2020), occurs one week before the appearance of robust social behavior (Dreosti et al., 2015; Larsch and Baier, 2018; Stednitz and Washbourne, 2020). *egr1*, in addition to being expressed in the pharyngeal pouch (PP; Fig. 1*A*), is predominantly expressed in a few clusters in the forebrain and midbrain. Given previous evidence for telencephalic contribution to social behavior (Stednitz et al., 2018), we noticed the prominent *egr1* expression in the telencephalon, including the BF, in cells adjoining the anterior commissure (AC; Fig. 1*B*). We conclude that *egr1* is expressed in a region that contributes to social behavior at a time when neuronal circuits are being established.

We obtained a mutant from the Sanger ZMP with a point mutation in the *egr1* gene (*sa64*). The C > A mutation generates a premature termination codon in exon 2, likely resulting in a truncated 137-aa protein. As this mutation is located upstream of the functional zinc finger DNA binding motifs (Fig. 1*C*), we predict that it results in loss of protein function. To eliminate possible additional background mutations, we used *egr1^sa64^* hets that had been outcrossed to wt zebrafish for seven generations.

Larvae derived from an incross of *egr1^sa64^* hets develop normally (Fig. 1*D*) and homozygous mutants were present at an approximate Mendelian ratio at 15 dpf (27.7 ± 13.6% SD; *N* = 4 clutches, *n* = 201 animals), a developmental time at which social behavior is already robust (Stednitz and Washbourne, 2020). However, we noticed a large proportion of larvae that were much smaller (< the mean minus 1.5 × the SD) than others, which we will call “stunted.” Mutants were significantly smaller than wt and hets on average (*p* = 0.002, *p* = 0.006), although stunted individuals were found across all genotypes and reached other developmental milestones with their siblings (Fig. 1*D*, bottom). For example, stunted larvae inflated their swim bladders by the same day (5 dpf) as all other animals, including homozygous *egr1^sa64^* mutants. Although the majority of homozygous *egr1* mutants were stunted (78 ± 12.9% SE across 8 clutches; Fig. 1*E*), almost half of the stunted larvae occurred in het and wt larvae (Fig. 1*F*). This phenotype is similar to the stunted growth reported in the mouse *Egr1* gene knock-out mutant (Topilko et al., 1998). Analysis of *egr1* using morpholine-modified oligonucleotides (MO) in zebrafish also resulted in reduced growth in morphants (Zhang et al., 2013). Therefore, despite the incomplete penetrance in our system, we conclude that *egr1* contributes to size of the animal. Because of the relatively high occurrence of the stunted phenotype in hets, we conclude that this phenotype may be because of a gene interaction with an, as yet, undetermined other gene. We also conclude that *egr1^sa64^* is likely to be a null allele, although we cannot exclude the possibility that it is a hypomorph. Importantly, we considered size as a factor for all additional experiments.

### Egr1 is necessary for social behavior

To examine social behavior in *egr1^sa64^* mutants of regular-sized and stunted animals, we used a split dyad assay with size-matched animals (Fig. 2*A*; Stednitz et al., 2018). Social behavior becomes robust at 14 dpf and is visually driven, with animals engaging in stereotypical orienting and approach behavior when they can see an animal of similar size (Dreosti et al., 2015; Stednitz and Washbourne, 2020). *egr1* mutants spent less time orienting at angles to the divider between 45° and 90° (*p* = 0.003; Fig. 2*B*), a strong indicator of social engagement (Stednitz et al., 2018), and less time close to the divider (Fig. 2*C*). Mutants were not less active during the assay period (*p* = 0.131; Fig. 2*D*). We conclude that Egr1 is involved in modulating social behavior.

Consistent with our previous findings on active reciprocation during social interactions in adult and larval zebrafish, wt and heterozygous fish paired with mutants also exhibited reduced social orienting (*p* = 0.003; Fig. 2*E*) and place preference (*p* < 0.001; Fig. 2*F*). Using multiple linear regression, we found that both the genotype of the target fish and the stimulus fish significantly influences orienting behavior (*p* = 0.021 and *p* = 0.040, respectively). Therefore, the deficit in social behavior of *egr1* mutants is detected by wt and heterozygous siblings. However, we also conclude that this social assay provides a variable social stimulus for test larvae that might lead to spurious results, especially with the added size effect.

**Figure 3.**
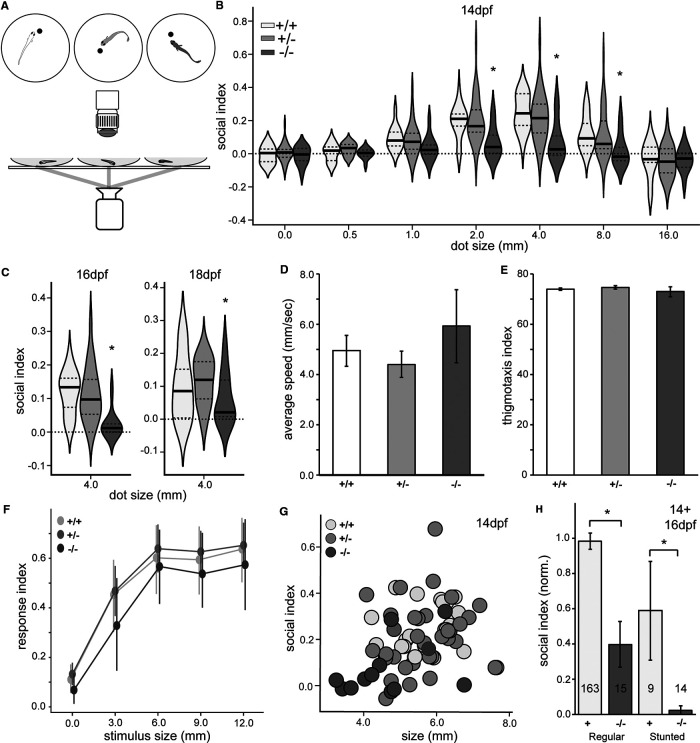
*egr1* mutants are less social with virtual stimuli than wt larvae. ***A***, Biological motion-based social behavior was assayed by projecting dots of different sizes to dishes from below, while recording with an IR camera from above. ***B***, Violin plots revealed significant differences for *egr1* mutants in SI for dot sizes of 2.0, 4.0, and 8.0 mm at 14 dpf. *N* = 3 experiments, *n* = 75 animals *p < 0.05, HSD, wt (+/+, white), het (+/−, light gray), mut (−/−, dark gray). ***C***, *egr1* mutants exhibited social deficits at 16 and 18 dpf, shown for optimal dot size 4.0 mm (dot size with highest SI response). *N* = 3 experiments, *n* = 129 animals, and *N* = 3 experiments, *n* = 97 animals, *p < 0.05, HSD. Plots of average speed in pixels per second (***D***) and thigmotaxis index (***E***) during biological-motion based assays at 4.0 mm dot size revealed no general behavioral deficits for *egr1* mutants. *N* = 3 experiments, *n* = 77 animals. ***F***, Mutant predator avoidance responses were not significantly different from het and wt siblings across various stimulus sizes at 14 dpf. *N* = 3 experiments, *n* = 126 animals. ***G***, Correlation plot of size (in pixels) and SI for 4 mm dot size at 14 dpf. *N* = 3 experiments, *n* = 75 animals. ***H***, The differences in SI for 4.0 mm dot size in 14 and 16 dpf larvae (normalized to average 14 dpf wt SI) between mutants and wt/hets (+, white) are significant in both regular-sized and stunted larvae. *n* = 201 animals, *p < 0.05, HSD. Error bars are SEM. Animal numbers are presented on bars.

To explore social engagement without stimulus variability, we tested whether a social deficit could also be detected using a virtual social assay that is based on detection of biological motion (Larsch and Baier, 2018). This assay reduces variability, as the stimulus is identical for each larva. Dots projected below dishes of solitary animals are followed preferentially when the dots move in bouts with kinetics similar to age matched animals (Fig. 3*A*; Larsch and Baier, 2018). Animal responses are tuned to dot size, with maximal social attraction at dot sizes approximating body size at 4.0 mm for 14 dpf larvae (Fig. 3*B*; Larsch and Baier, 2018). Larger dots (16 mm) elicit an aversive response, whereas small dots (0.5 mm) are ignored and are equivalent to no dot (0.0; Fig. 3*B*). Homozygous *egr1^sa64^* mutant larvae are less attracted to visual stimuli 2.0, 4.0, and 8.0 mm in diameter than wt siblings (*p* = 0.0086, 0.0034, 0.047, HSD; Fig. 3*B*). This reduction in social behavior is also evident at 16 and 18 dpf (*p* < 0.0001, *p* = 0.009, HSD), suggesting the deficit is not a short developmental delay that is recuperated over 4 d of development (Fig. 3C). *egr1* genotype does not affect animal swimming speed or thigmotaxis (*p* = 0.69, 0.76, HSD; Fig. 3*D*,*E*). Furthermore, predator avoidance responses to a looming stimulus are not significantly different between all genotypes at 14 dpf (*p* > 0.25; HSD; Fig. 3*F*), suggesting that vision is not accountable for the reduction in SI. We conclude that Egr1 is necessary for following biological motion in a social context, but not for visual and motor system functions.

We considered that the growth deficit (Fig. 1*D–F*) might account for the reduction in SI. We detected a weak, yet significant, correlation between animal size and SI (*R* = 0.37, *R*^2^ = 0.14, *p* = 0.0011; Fig. 3*G*). This effect was consistent across individual genotypes and at all ages examined. We further explored the influence of size by comparing the SI of animals in groups of similar size. Consistent with previous observations (Larsch and Baier, 2018), larger wt and heterozygote animals have a 67% higher SI than wt stunted animals (*p* = 0.186, HSD, *n* = 9 stunted animals; Fig. 3*H*, +). However, mutants exhibited reduced social behavior both for regular-sized and stunted individuals (*p* = 0.0001, HSD; Fig. 3*H*, −/−). We conclude that genotype, rather than size, is the primary factor influencing social behavior deficits in *egr1* mutants.

### Egr1 is necessary for TH2 expression

**Figure 4.**
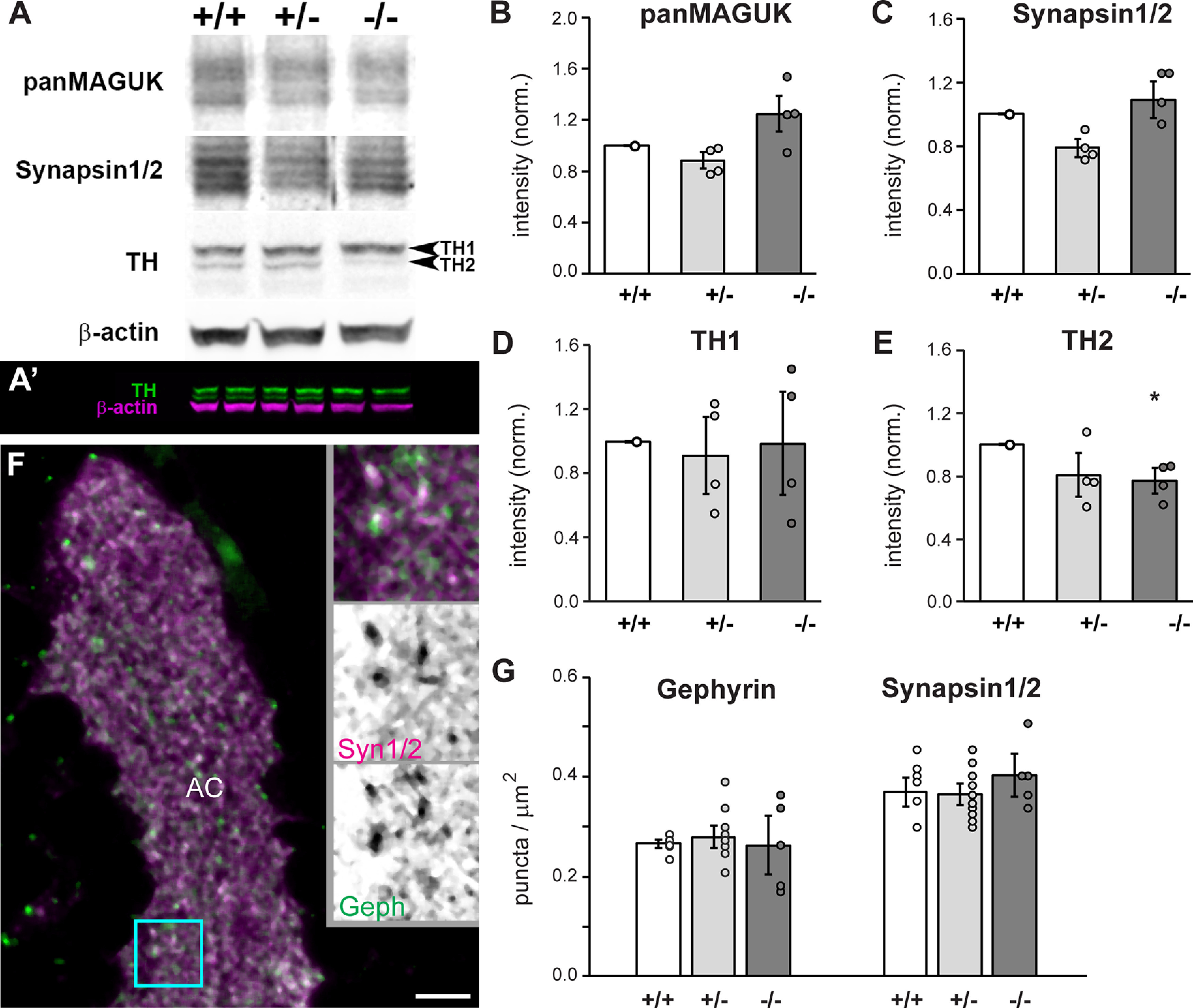
Egr1 is necessary for TH2 expression. ***A***, Western blotting of larval head homogenates at 14 dpf reveals a reduction in expression of TH2 in *egr1* mutants. ***A’***, Two color labeling of a Western blotting to highlight in-lane normalization to β-actin and technical replicates. ***B–E***, Western blot intensities in ***A***, ***A’*** were first normalized to in-lane β-actin, and then to wt average intensity. *N* = 4 experiments, *n* = 2 technical replicates, *p < 0.05, HSD. ***F***, Immunolabeling of synapses with antibodies to Synapsin 1 and 2 (Syn1/2, magenta) and Gephyrin (Geph, green) in the anterior commissure (AC), lateral view. Insets are magnifications of the cyan box. Scale bar: 5 μm. ***G***, Quantification of synaptic puncta revealed no changes in synapse density in the medial and lateral AC. *n* ≥ 5 brains per genotype. wt (+/+, white), het (+/−, light gray), mut (−/−, dark gray). Error bars are SEM.

The Egr1 transcription factor regulates expression of proteins that play a role in synaptic transmission and NT synthesis (Duclot and Kabbaj, 2017). We used Western blotting of protein extracts from 15 dpf larval heads to explore whether Egr1 promotes social behavior by influencing abundance of such proteins in the brain. Levels of MAGUK proteins (panMAGUK), such as PSD-95, SAP102 and SAP97, and Synapsin proteins 1 and 2 (Synapsin 1/2) were not significantly influenced by *egr1* loss of function (*p* > 0.8, HSD; Fig. 4*A–C*). In contrast, an antibody that recognizes two forms of TH, which based on predicted molecular weights likely correspond to TH1 and TH2, revealed that levels of TH2 but not TH1 are compromised by mutation in *egr1* (TH1: *p* = 0.9, TH2: *p* = 0.0485, HSD; Fig. 4*A*,*A’*,*D*,*E*).

Given the bias of Egr1 toward regulating synaptic genes (Duclot and Kabbaj, 2017), we assessed whether Egr1 influences forebrain synapse abundance. We quantified synapses by immunolabeling sections of 15 dpf larval brains with antibodies to Synapsin 1/2 and Gephyrin in *egr1^sa64^* mutants (Fig. 4*F*). Synapsins are enriched at over 95% of vertebrate synapses (Micheva et al., 2010), whereas Gephyrin is a specific marker of inhibitory synapses (Fritschy et al., 2008). Within the BF, a region with high expression of *egr1* (Fig. 1*A*,*B*) and a region implicated in controlling social behavior (Stednitz et al., 2018), we focused on the neuropil of the AC. Quantification of the number of puncta, an approximation of synapses, in the medial and lateral AC neuropil showed no difference in total (Synapsin 1/2, *p* = 0.71, HSD) or inhibitory (Gephyrin, *p* = 0.99, HSD) puncta density in *egr1* mutants compared with wt (Fig. 4*G*). The lack of effect on synapse number is consistent with the Western blotting results above, which demonstrated that levels of the widespread synapse markers Synapsin 1/2 and PSD-95 are unchanged in *egr1* mutants. However, our results do not preclude the possibility that specific synapse populations might be regulated by Egr1. We conclude that Egr1 regulates the abundance of TH2 protein, but does not regulate presynaptic or postsynaptic protein abundance of Synapsin 1/2 or MAGUK proteins, nor general synapse density.

**Figure 5.**
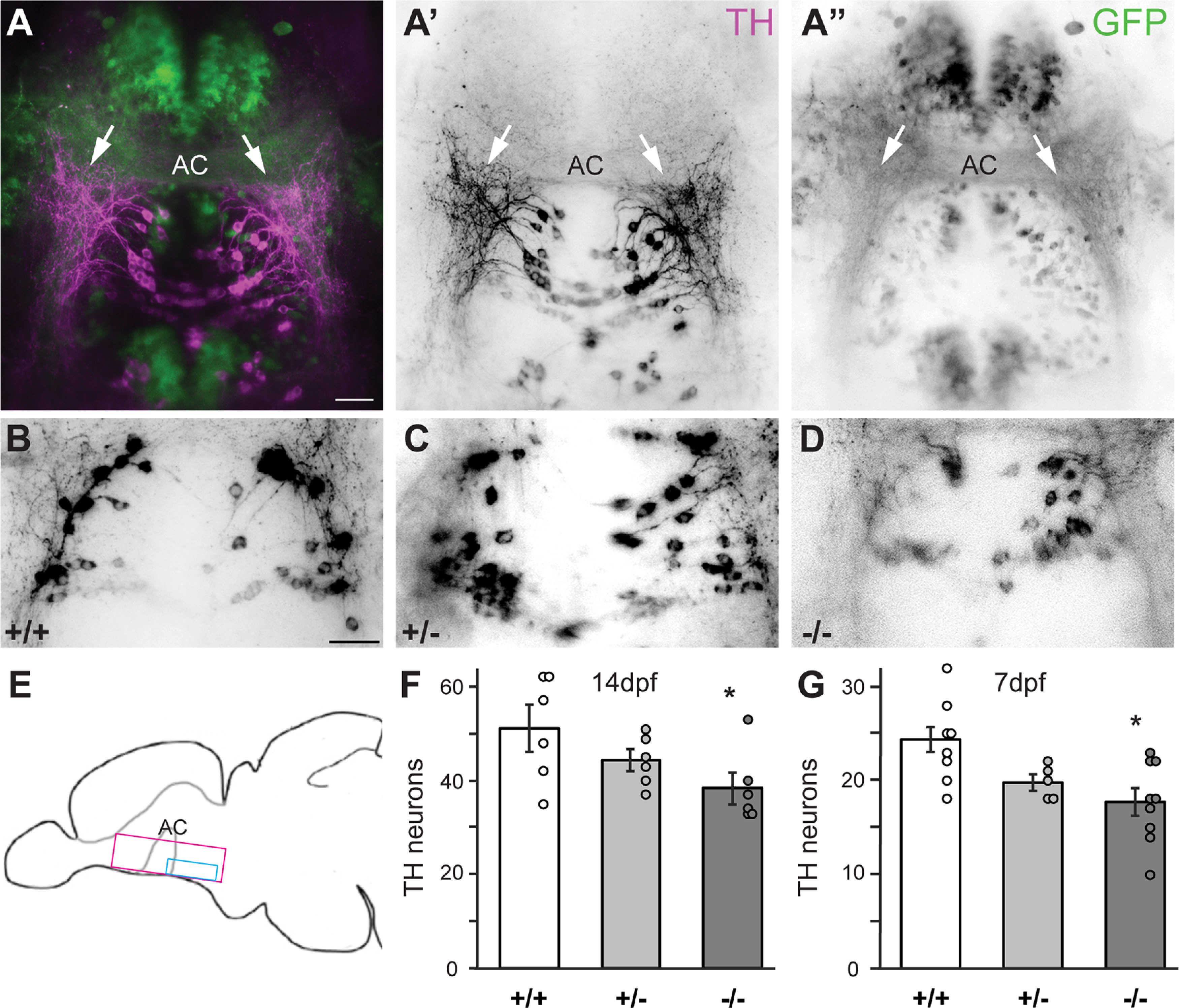
Egr1 is necessary for TH-positive neuron number in BF. ***A–A’’***, Dorsal view of maximum projection of the BF region (magenta box in ***E***) of *Et ^y321^* larval zebrafish at 14 dpf labeled with antibodies to GFP (green) and TH (magenta). Anterior commissure (AC); anterior is to the top. ***B–D***, Maximum projections of TH labeling in the BF region posterior to the AC for *egr1* wt (***B***), hets (***C***) and mutants (***D***). Scale bars: 30 μm. ***E***, Diagrammatic side-view of the larval brain highlighting the regions (magenta and cyan) analyzed as a maximum projection Z-stacks in ***A***, ***B–D***, respectively. ***F***, Quantification of the number of TH-positive neurons in the cyan region in E revealed a decrease in *egr1* mutants at 14 dpf. *N* = 2 experiments, *n* = 18 brains, *p* < 0.05 (HSD). ***G***, The same quantification as in F at 7 dpf demonstrates a consistent decrease in TH-positive neurons during development. *N* = 2, *n* = 24, *p* < 0.05 (HSD). wt (+/+, white), het (+/−, light gray), mut (−/−, dark gray). Error bars are SEM.

A synaptic connection between TH-positive neurons and cholinergic BF neurons (cBFNs) defined by the enhancer trap line *Et ^y321^* [*Et(rex2-scp1:gal4ff)y321*] was recently identified (Goode et al., 2020). Chemogenetic ablation of cBFNs disrupts social attraction and orienting behavior (Stednitz et al., 2018). Images from the most basal region of the forebrain (Fig. 5*E*), including the anterior part of the presumptive PPa, reveal that the TH-positive neurons project to the AC neuropil (Fig. 5*A–A’’*, white arrows), which is also innervated by cBFN arbors (Goode et al., 2020). TH-positive neurons in the PPa are highly likely to be dopaminergic (Yamamoto et al., 2011), so we will refer to these cells as dPPaNs. Cells in this region have been documented to express both *th1* and *th2* genes in larvae and adults (Semenova et al., 2014).

We explored whether TH expression in dPPaNs requires Egr1 (Fig. 5*B–D*). The number of TH-positive dPPaNs was significantly reduced in homozygous *egr1^sa64^* mutants relative to wt siblings at 14 dpf (*p* = 0.049, HSD; Fig. 5*F*). The ∼28% reduction of TH-positive dPPaNs in mutants was evident as early as 7 dpf (Fig. 5*G*), a time at which stunted individuals were not detected. Interestingly, we found an intermediate, but not significant, phenotype in TH cell number in heterozygous *egr1^sa64^* mutants as compared with wts at both time points (*p* = 0.362, *p* = 0.099, HSD), analogous to results from the Western blot experiments (Fig. 4*E*). This may indicate a haplo-insfficiency at the molecular level, although this is not detected at the behavioral level. Together, our results suggest that the discrepancy in TH expressing cells is established early in development, consistent with the expression of *egr1* in the BF at this developmental stage (Fig. 1*B*). Given the expression of both *th1* and *th2* in dPPaNs (Semenova et al., 2014), we conclude that our result is because of a decrease in the actual number of dPPaNs, and not because of a detection limit as a consequence of a *th2* expression decrease. Our experiments suggest that catecholaminergic, presumably dopaminergic, signaling in this part of the larval brain is compromised by loss of Egr1, although it is possible that feedback mechanisms might compensate for the NT deficit.

### BF TH neurons are necessary for social behavior

**Figure 6.**
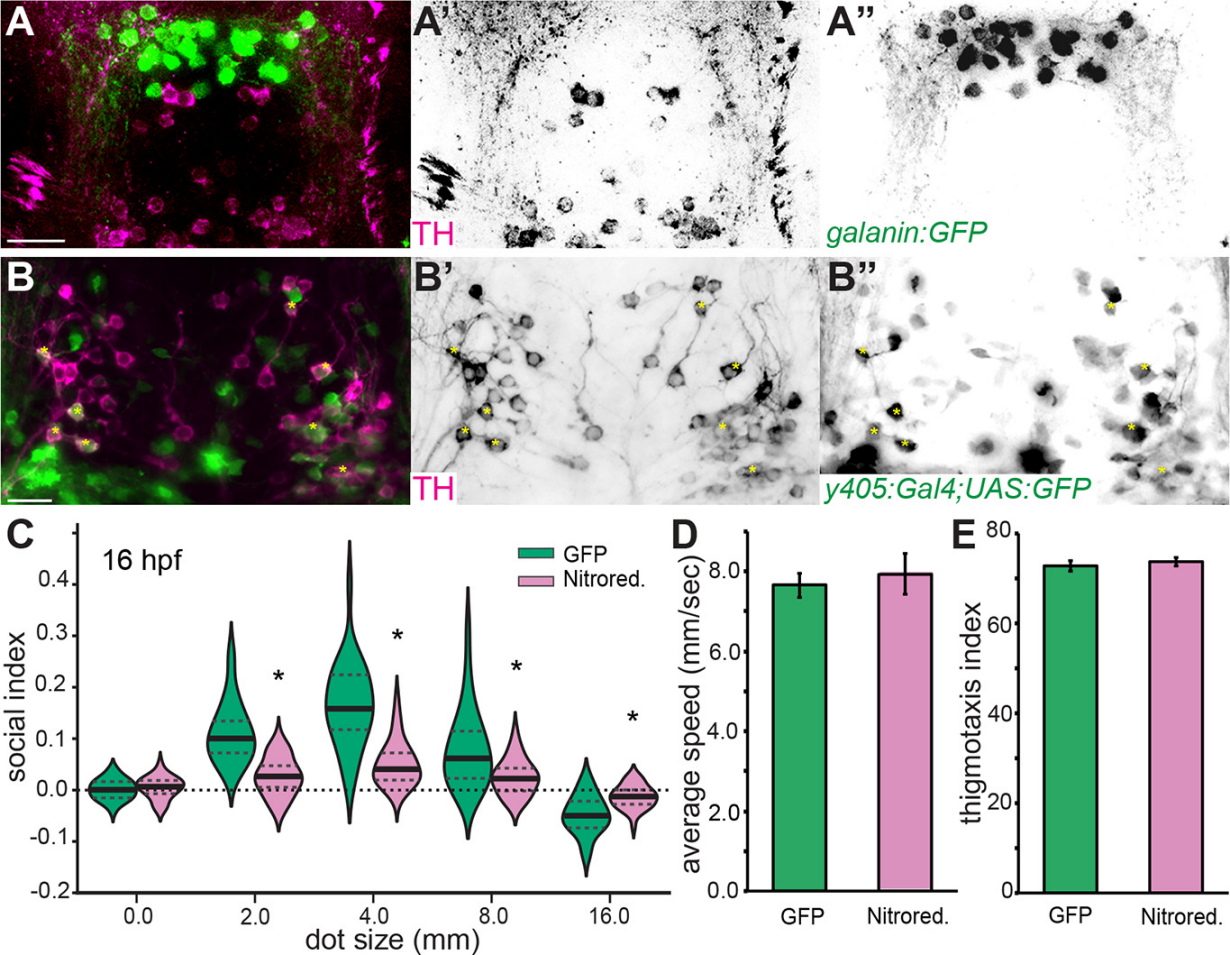
TH-positive neurons in PPa are necessary for social behavior. ***A–A’’***, Maximum projection of the PPa of 14 dpf *Tg(galanin:GFP)* larvae immunolabeled for GFP (green) and TH (magenta) revealed two distinct populations. Dorsal view of cyan box in Figure 5*E*, anterior to the top. ***B–B’’***, Maximum projection of PPa of 14 dpf *Et ^y405^* larvae immunolabeled for GFP (green) and TH (magenta) revealed transgene expression in ∼30% of TH-positive neurons (yellow asterisks). Scale bars: 20 μm. ***C***, Violin plots of SI for *Et ^y405^* larvae with nitroreductase-ablated neurons at 16 dpf (magenta) revealed significant differences compared with control larvae only expressing GFP (green). Both groups were treated with nifurpirinol. *N* = 2 experiments, *n* = 60 animals, *p < 0.001, *t* test comparisons to GFP control. Plots of average speed in pixels per second (***D***) and thigmotaxis index (***E***) during biological-motion based assays at 4.0 mm dot size revealed no general behavioral deficits for larvae with *Et ^y405^* neurons ablated. *N* = 2 experiments, *n* = 60 animals, *p* > 0.5. Error bars are SEM.

To gain genetic access to the TH-positive neuron population and test a possible role for these neurons in social behavior, we examined driver lines that express in the BF. The *Tg(galanin:GFP)* line expresses in neurons in the PPa, and the neuropeptide galanin has been implicated in various mammalian social behaviors (Wu et al., 2014). Labeling of *galanin:GFP* transgenic animals with TH antibody revealed that galanin-expressing and TH-positive neurons are distinct populations (Fig. 6*A*). The enhancer trap line *Et ^y405^* [*Et(scp1:gal4ff)y405*], in which the construct integrated in the *robo2* gene, also drives expression in the PPa. In contrast to the galanin driver, ∼30% of neurons labeled by the *Et ^y405^* driver overlapped with TH-positive dPPaNs (29.2 ± 5.1%, *N* = 3 brains; Fig. 6*B*).

**Figure 7.**
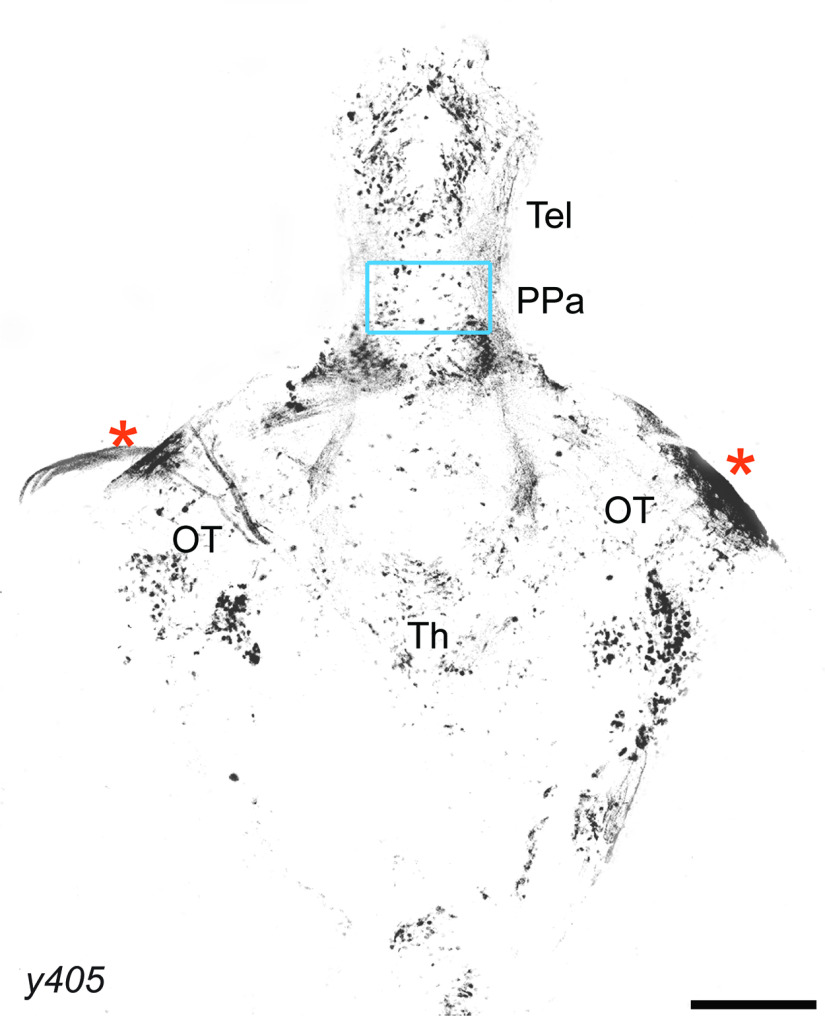
Whole-brain expression of *Et ^y405^* at 16 dpf. Maximum Z projection of *Et ^y405^ (y405)* brain expressing GFP, with the region of interest from Figure 6*B* marked with a cyan box. Anterior to the top; * nonspecific labeling of meninges. Tel, telencephalon; PPa, anterior parvocellular POA; OT, optic tectum; Th, thalamus. Scale bar: 200 μm.

We hypothesized that chemogenetic ablation of neurons marked by *Et ^y405^* would reduce dPPaNs similar to *egr1* loss of function, allowing us to test whether a reduction of dPPaNs might be the cause of reduced social behavior in *egr1* mutants. We treated larvae generated by a cross between the *Et ^y405^* driver and a nitroreductase expression line with the antibiotic nifurpirinol. The enzymatic activity of nitroreductase on nifurpirinol generates a toxic by-product that kills cells in a cell-autonomous fashion. Indeed, treatment of animals with nifurpirinol resulted in a 31% reduction in dPPaNs when the nitroreductase transgene was expressed (GFP control: 82 neurons ± 2.5, nitroreductase: 56.2 ± 5, *n* = 11 brains, *p *<* *0.005, *t* test). Biological motion-based social behavior was significantly reduced in larvae with ablated dPPaNs compared with control animals at dot sizes 2.0, 4.0, 8.0, and 16.0 (*p* < 0.001, *t* test; Fig. 6*C*). No deficits were detected for swimming speed or thigmotaxis (*p* > 0.5, *t* test; Fig. 6*D*,*E*). Chemogenetic ablation of random neuronal populations in larvae does not always cause a deficit in social behavior (Stednitz et al., 2018), suggesting that the social behavior deficit is specific to the ablated population and not because of general neurologic trauma. We cannot exclude that ablation of neurons in other regions of the brain within the *Et ^y405^* line might contribute to the social deficit, as the *Et ^y405^* enhancer trap drives sparse expression throughout the brain at 16 dpf (Fig. 7). However, our results are consistent with the conclusion that dPPaNs are necessary for robust social behavior.

## Discussion

In this study, we examined the contribution of *egr1*, a gene implicated in the etiology of neuropsychiatric disorders, to the establishment of social behavior in zebrafish. Egr1 regulates social behavior as measured by two different assays: a split dyad assay (Fig. 2) and a biological motion-based assay (Fig. 3). Investigation of downstream proteins that might be affected by a loss of this transcription factor revealed a decrease in TH2 protein, but no differences in other known targets of Egr1 (Fig. 4). We examined a population of TH-positive neurons in the PPa that are in synaptic contact with identified “social” neurons of the BF and discovered a reduction in the number of the TH-positive neurons in *egr1* mutants (Fig. 5). Chemogenetic ablation of a similar number of TH-positive neurons in the PPa resulted in a comparable reduction in social behavior (Fig. 6).

Our findings using *egr1^sa64^* suggest two important conclusions: (1) Egr1 is implicated in regulating social behavior, and (2) dPPaNs represent a novel population of neurons implicated in driving social behavior. We discuss these findings further below.

First, the behavioral deficit in *egr1* mutants appears to be relatively specific to social behavior, as it does not affect vision or general motility. This specificity may lie in the *egr1* expression pattern. From the first day of development, *egr1* is specifically expressed in the ventral telencephalon and diencephalon (Close et al., 2002). This expression is puzzling for an IEG, as it appears long before one would predict high levels of neuronal activity. In fact, activity in the BF social circuit presumably starts during the second week of development, as this is when social behavior becomes apparent (Stednitz and Washbourne, 2020). Interestingly, Egr1 has been described to act as a developmental transcription factor in some tissues, for example during development of skeletal muscle (Zhang et al., 2018). Therefore, it is possible that a primary role of Egr1 in the BF is activity-independent regulation of TH expression in dPPaNs. In the absence of *egr1* expression, dPPaNs presumably synthesize and release less dopamine, reducing critical input to cBFNs and a putative social network in the BF, resulting in aberrant social behavior. Our finding is reminiscent of the loss of another IEG, *FosB*, in mammals. Most behaviors are largely the same in *FosB* knock-out mice, suggesting no ongoing deficit in neuronal function across most of the brain. However, nurturing of neonates is almost absent in knock-out dams, which was explained by deficient signaling in the POA (Brown et al., 1996). This may suggest an intriguing role for IEGs in establishing circuitry and/or cell type during development, and in the cases of Egr1 and FosB, particularly within social brain networks.

Indeed, the effect we describe in zebrafish induced by the *egr1* mutation is consistent with the identification of *EGR1* as a susceptibility gene for neuropsychiatric disorders including schizophrenia and depression (Covington et al., 2010; Kimoto et al., 2014; Cattane et al., 2015). Our results also bolster the idea that *EGR1* is a hub for other genes involved in synaptic transmission that are affected in ASD (Liu et al., 2016). Although EGR1 levels are altered in various disorders including schizophrenia (Yamada et al., 2007), it is important to note that mutations in the human population have not yet been identified which directly link EGR1 and these disorders. It is possible that in humans *EGR1* mutations are severe or lethal, and that only other mutations or environmental influences that modulate EGR1 activity or expression levels result in neuropsychiatric symptoms. This postulated exclusive mode of EGR1 modulation via other effectors may be unique to humans, as the gene has been implicated in a human-only network of genes aberrant in ASD cases (Liu et al., 2016).

Second, our study of the molecular mechanisms underlying the social phenotype of *egr1* mutants implicates a population of neurons, dPPaNs, and their connections in driving social behavior. The PPa region lies within the POA, an integral part of the conserved vertebrate social behavior network (O’Connell and Hofmann, 2012). Consistent with this categorization, the PPa was recently implicated in zebrafish social behavior. Developmental oxytocin-cell ablation, which reduces social behavior, eliminates activation of the PPa by social stimuli (Nunes et al., 2021). Our results now define a precise population of cells within the PPa that play a role in controlling social approach and orienting. Our previous finding that TH-positive terminals synapse on cBFN^y321^ arbors suggests that the dPPaNs identified here are presynaptic to cBFNs (Goode et al., 2020). It is yet more intriguing to consider that these terminals increase their Synaptotagmin 2a protein levels when social behavior becomes robust at 14 dpf (Goode et al., 2020), implicating a developmental gain of function in the dPPaNs as the trigger that “turns on” social behavior. This insight now ties two concrete neuronal populations together as a first step in elucidating a social circuit at molecular and cellular resolution.

Finally, based on anatomic marker studies of the developing zebrafish brain, the TH-positive PPaNs are most probably dopaminergic (Yamamoto et al., 2011; Gunaydin and Deisseroth, 2014). The putative dopaminergic identity of dPPaNs raises interesting possibilities, as the dopamine system is affected in neuropsychiatric disorders with social components, such as ASD (Hettinger et al., 2008). Dopamine is generally considered to be part of a reward system; therefore, dPPaNs could be part of a reward circuit for social motivation (Gunaydin and Deisseroth, 2014). Alternatively, dPPaNs could also modulate social cognition (Gunaydin and Deisseroth, 2014). Future studies to discriminate between these two options will be critical for understanding the circuits and mechanisms underlying social approach and orienting behavior. Although further transcriptomic characterization will help to define the molecular identity of dPPaNs, our findings already inform potential pharmacological intervention strategies for the social component of neuropsychiatric disorders.

## Synthesis

Reviewing Editor: Benjamin Deneen, Baylor College of Medicine Decisions are customarily a result of the Reviewing Editor and the peer reviewers coming together and discussing their recommendations until a consensus is reached. When revisions are invited, a fact-based synthesis statement explaining their decision and outlining what is needed to prepare a revision will be listed below. The following reviewer(s) agreed to reveal their identity: Marco Vindas, Dan Gorelick.

The reviewers found this work to be of very high quality and are generally supportive of publication with a few revisions that are well within your grasp. Specifically, there were a few major issues brought to light by Reviewer 2 that need to be addressed prior to publication. These include additional evidence that sa64 is null allele and the nature of the observed stunted phenotype. If, in fact, sa64 is a hypomorph rather than a null allele, then this needs to be reflected in the language used in the manuscript. Finally, TH2 staining was not convincing and needs to be improved. Regarding the comments from reviewer 1, these mainly involve phrasing and clarification of data, which should also be addressed in your response and in any revised version of the manuscript.

Reviewer comments

Reviewer 1

This manuscript involves a series of experiments and techniques used to investigate the role of EGR1 in the regulation of TH neurons and the possible role this has in social behavior in zebrafish. The experiments and techniques are robust and well established. In general, the manuscript provides most of the information in a clear and concise manner. I have only some minor comments and concerns.

1. Ln 104-140: Behavioral assays: it would be great if the authors could include the total number of fish that were tested in each behavioral test for each genotype

2. Ln 115-116: the authors write that larva were individually tested at the ages indicated, but I don't understand what they mean by this. Where are these ages indicated? Later in line 123 they write that the larvae were tested only at 14 dpf, could the authors clarify this?

3. Ln 114-124: Biological motion social assays: it would be better if the authors include here a description of what this assays measures. They include this explanation later in the results section, but in my opinion, this should be better described in this section.

4. Ln 132-140: Predator avoidance test: can the authors include the total time for which fish were tested for?

5. Ln 201: Can the authors include a reference for their statement that one week prior to 6 dpf zebrafish show robust social behavior? This is the first time this is named in the manuscript and the reference should therefore be provided here. Similarly, a reference for their statement that circuits are being formed at 6 dpf is also missing and a reference for egr1 predominantly being expressed in the forebrain and midbrain.

6. Ln 218, 237, 239, 267: please include the p values here.

7. Ln 259: please tone down your statement that your results are evidence suggesting the deficit is not a developmental delay that is recuperated over time, since this is only tested over a 4 dpf difference and it is still possible that the developmental delay may be recuperated over a longer time.

8. Ln 263: please include an explanation of what the social index is and how it is calculated

9. Ln 271: I am confused, larger wt and heterozygote animals have a 67% higher SI than wt stunted animals, but their p value (0.186) is not significant... is there a mistake here?

10. Ln 308: include reference for AC neuropil being innervated by cBFN arbors.

11. Ln 319: the authors have not evaluated if dopaminergic signaling is in fact reduced in these animals. They have only found a decrease in TH neurons. There might be a compensatory mechanism at play here with an increase in signaling due to upregulation in for example DA receptors or inhibition of autoreceptors. One should corroborate that DA levels are diminished in these fish by for example by quantification of monoamine neurochemistry.

12. Ln 331-348: the authors have found that it is TH2 which is reduced in mutants, but when they test the reduction in TH-positive dPPaNs they don't distinguish between TH1 and TH2. This would be a key component to include in their studies particularly considering that TH2 is exclusively expressed in specific neuronal populations in the forebrain and midbrain (see Semenova, S. A., Chen, Y. C., Zhao, X., Rauvala, H., Panula, P. (2014). The tyrosine hydroxylase 2 (TH2) system in zebrafish brain and stress activation of hypothalamic cells. Histochemistry and cell biology, 142(6), 619-633).

13. Ln 361-362: I don't completely agree with their statement: "Egr1 is implicated in specifically regulating social behavior". They haven't studied all possible functions by EGR1 so this is a bit of an overstatement.

14. Ln 373: similarly to on comment 11, the authors haven't actually tested if DA synthesis is actually reduced or not, it is very likely yes, but it hasn't been quantified, so please tone down this statement

15. Figure 2 (Ln 538): what the authors mean by relative place preference. This should be clear in the figure text.

Reviewer 2

The manuscript reports that Egr1 sa64 homozygous zebrafish mutants exhibit a behavioral phenotype and conclude that erg1 regulates social behavior. These conclusions appear supported by the data, although the manuscript would be more rigorous if the authors could re-analyze the data to should show direct correlation between number of neurons and behavior phenotype in the ablation studies. I'm not convinced that sa64 is a null mutant, it is possibly a hypomorph. The stunted phenotype has variable penetrance. Additionally, the TH2 expression phenotype is not convincing. This doesn't change the conclusions from the behavior and ablation studies. Unless the authors can provide more support for their claims regarding a null mutation, it might be prudent for the authors to tone down the language rather than write that sa64 is likely to be a null allele.

Specific comments:

Please provide more detail about the stunted phenotype in sa64 zebrafish, to support claims that this allele is a null mutation. Do stunted fish ultimately reach normal length, just grow more slowly than normal fish? Or are the fish permanently stunted? There appears to be an increase in the stunted phenotype in sa64 heterozygous fish vs wildtype (fig 1). What accounts for this? Is something similar observed in mouse Egr1 heterozygotes? The sa64 homozygous stunted phenotype exhibits incomplete penetrance. Is this also the case in mouse Egr1 homozygous mutants (Topilko et al, 1998)? For Fig 1E, 1F, it might be helpful to visualize the sa64 phenotype distribution per clutch, in addition to overall - is the percent larvae with the stunted phenotype the same in each clutch? Would also be helpful to see individual data points in Fig 1E, 1F.

Fig 4. Data showing reduction of TH2 in sa64 -/- are not convincing. To my eye, there appears to be a similar reduction in synapsin1/2 in sa64 hets vs wt (and vs homozygotes) (Fig 4A), yet this is not statistically significant (Fig 1C)? Quantifying protein amounts using western blots is fraught with inaccuracies, and it's not clear whether the manuscript accounted for issues such as proper standards for normalization, determining the linear range for the antibodies/detection system, etc (see PMID 25059473). For all data in Fig 4, it would be helpful to see individual data points on each bar graph.

Line 314: "Interestingly, we also found an 18%, but not significant, reduction in TH cell number in heterozygous egr1sa64 mutants as compared to wts (p = 0.0985 HSD)." I'm not sure what the authors mean by this. My interpretation is that there is no reduction in TH cell number in sa64 heterozygotes vs wildtype. Is this accurate?

Fig 6. Can the authors please provide a correlation for the social index/average speed/thigmotaxis and the number of dPPaNs following nitroreductase treatment? Line 338 provides data on average number of neurons in control vs nitroreductase fish, but it would be helpful to see direct correlation between, for example, dot size 4.0 mm and social index GFP together with number of dPPaNs (and the same for 4.0mm and nitroreductase, etc). To support the conclusion, the manuscript should show that reduced social index correlates with reduced number of dPPaNs.
